# LAMP2A, and other chaperone-mediated autophagy related proteins, do not decline with age in genetically heterogeneous UM-HET3 mice

**DOI:** 10.18632/aging.204796

**Published:** 2023-06-13

**Authors:** Katherine K. Zhang, Peichuan Zhang, Anagha Kodur, Ilkim Erturk, Calvin M. Burns, Cynthia Kenyon, Richard A. Miller, S. Joseph Endicott

**Affiliations:** 1 University of Michigan, College of Literature, Science, and The Arts, Ann Arbor, MI 48109, USA; 2 Calico Life Sciences, South San Francisco, CA 94080, USA; 3 Current Affiliation: WuXi AppTec, Shanghai, China; 4 University of Michigan, Department of Pathology, Ann Arbor, MI 48109, USA; 5 University of Michigan Geriatrics Center, Ann Arbor, MI 48109, USA

**Keywords:** chaperone-mediated autophagy, autophagy, aging

## Abstract

Chaperone-mediated autophagy (CMA) selectively degrades proteins that are crucial for glycolysis, fatty acid metabolism, and the progression of several age-associated diseases. Several previous studies, each of which evaluated males of a single inbred mouse or rat strain, have reported that CMA declines with age in many tissues, attributed to an age-related loss of LAMP2A, the primary and indispensable component of the CMA translocation complex. This has led to a paradigm in the field of CMA research, stating that the age-associated decline in LAMP2A in turn decreases CMA, contributing to the pathogenesis of late-life disease. We assessed LAMP2A levels and CMA substrate uptake in both sexes of the genetically heterogeneous UM-HET3 mouse stock, which is the current global standard for the evaluation of anti-aging interventions. We found no evidence for age-related changes in LAMP2A levels, CMA substrate uptake, or whole liver levels of CMA degradation targets, despite identifying sex differences in CMA.

## INTRODUCTION

Protein turnover via the lysosome/autophagy system is a crucial regulator of aging [1–6]. Lysosomal proteolysis in mammalian cells occurs through at least three distinct pathways: macroautophagy (MA), microautophagy (mA), and chaperone-mediated autophagy (CMA). Each branch of autophagy is regulated independently, and each is responsible for degrading a different subset of intracellular cargoes. Drug treatments or genetic modifications that enhance macroautophagy reliably extend the lifespans of invertebrates [7].

Studies of autophagy and aging in mouse models are not yet as comprehensive as those in invertebrates. However, a few mouse studies do suggest a strong link between MA and longevity. The first study to show that genetic activation of macroautophagy could extend lifespan in mice was published in 2013, when Pyo et al. showed that two strains of C57BL/6J mice overexpressing ATG5 (ATG5 TG) had a 17% increase in median lifespan, with no sex differences [8]. ATG5 TG mice develop normally, show no differences in food intake, and have normal memory (as evaluated by Y-maze tests) [8]. However, ATG5 TG mice have reduced adiposity, increased insulin sensitivity, increased oxygen consumption rates, and reduced circulating leptin levels [8]. At 6 and 12 months of age, ATG5 TG mice have improved performance on wire hang tests [8].

Additional support for the role of macroautophagy in extending healthy lifespan in mice comes from inbred C57BL/6J mice with a homozygous F121A knock-in mutation to the gene encoding BECN1 (*becn1*^F121A/F121A^). The autophagy inhibitor BCL2 binds to BECN1, reducing its ability to nucleate phagophore formation. The F121A mutation reduces the interaction between BECN1 with BCL2, boosting the initial step of macroautophagy. BECN1^F121A/F121A^ mice have significantly extended median lifespan (roughly 11% for females and 12% for males), have a significant reduction in the formation of neoplastic lesions, and show reduced signs of age-associated degeneration in multiple tissues [9].

While it is clear that boosting MA in C57BL/6J mice is sufficient to extend lifespan, the relationship between CMA and lifespan is far less clear. Many review papers claim that CMA decreases with age in most cell types and tissues of mice and rats (key results are summarized below) [4, 10–13]. According to this paradigm, the main factor responsible for the age-related decline in CMA is a reduction in LAMP2A, the main component of the CMA substrate translocation complex. The abundance of the *Lamp2a* transcript is not changed with age [14, 15]. Rather, the stability of the LAMP2A protein at the lysosome is reduced, which is attributed to an age-related change in lysosomal membrane lipid composition [14, 16, 17]. While most papers reporting changes in CMA with age have focused on age-related declines in LAMP2A protein levels, one paper identified an age-related decline in HSPA8 in skeletal muscle of male C57BL/6J mice [18]. HSPA8 (also known as HSC70 or HSC73) is the chaperone that recognizes the pentapeptide consensus motif present in proteins targeted for degradation by CMA [19, 20].

There is compelling evidence that CMA degrades proteins whose overaccumulation contributes to age-associated pathologies, including fatty-liver disease, atherosclerosis and cancer [21–23]. Moreover, CMA is responsible for regulating the abundance of many well-studied proteins associated with neurodegeneration, including SNCA, MAPT, HTT, APP, LRRK2, and TARDBP [1, 24–28]. Rats with reduced CMA in the brain exhibit death of dopaminergic neurons in the substantia nigra and display Parkinson’s disease like motor symptoms [29]. Because of the clear link between CMA and age-associated diseases, it has been hypothesized that an age-related decline in CMA might contribute to the pathogenesis of age-associated morbidities [1, 10, 13].

An age-related decline in CMA was first reported over 20 years ago, when isolated liver lysosomes from 20-hour fasted 22-month-old male Fisher-344 rats were found to have reduced uptake of CMA substrates compared to lysosomes from 3-month-old animals [30]. The lysosomes from old animals were reported to show increased levels of lumenal proteins, such as cathepsins and hexosaminidase [30]. Both whole liver lysates and liver lysosomes from male FVB mice at 22 months of age have sharp reductions in LAMP2A levels, compared to samples derived from 6-month-old controls. Lysosomes from 22-month-old animals also showed significantly less uptake of GAPDH than lysosomes from 6-month-old animals [31].

Evidence for age-related decline in CMA has also been reported in tissues other than the liver. Hematopoietic stem cells (HSCs) from male C57BL/6J mice expressing a KFERQ-Dendra2 CMA reporter showed an age-related decline in CMA, with HSCs from 30-month-old animals showing a reduction in KFERQ-Dendra2 puncta compared to 4- and 12-month-old animals [32]. CMA was induced in CD4+ T cells isolated from 4-month-old C57BL/6J mice of unspecified sexes, by stimulating them with anti-CD3 and anti-CD28 [15]. However, this induction failed to occur in CD4+ T cells isolated from 22-month-old animals [15].

Contrary to reports that LAMP2A (and CMA) decreases in some tissues and cell types with age, other papers have reported evidence of increased CMA with age. The retinas of 22-month-old and 12-month-old C57BL/6J mice of unspecified sexes showed reduced LC3 flux (indicating reduced macroautophagy) compared to retinas from 3-month-old animals [33]. Despite the reduction in LC3 flux, metabolic labeling experiments found that there is increased turnover of long-lived proteins in retinas of 22-month-old animals compared to 3-month-old animals [33]. This increased protein turnover might be attributable to CMA, because retinas from 12-month-old and 22-month-old animals have higher expression of LAMP2A than retinas from 3-month-old animals [33]. Male C57BL/6J mice have reduced protein levels of LAMP2A and HSPA8 in skeletal muscle in old age (24-29 months), compared to young mice (3.5-7 months) [18]. However, the same mice have increased expression of LAMP2A in the heart in old age, with no change in HSPA8 [18]. Although simply measuring the abundance of LAMP2A and HSPA8 cannot conclusively indicate a change in CMA, the findings that LAMP2A levels increase in the retina and cardiac muscle with age stand in contrast to the paradigm that an age-related decline in LAMP2A levels causes a decrease in CMA.

Independent from its possible role in degrading disease-associated and neurodegeneration-associated proteins, CMA might modulate aging through other pathways. Recently published evidence supports the hypothesis that sustained activation of CMA in early and mid-life might promote longevity and good health by reducing the abundance of proteins that accelerate the pace of aging. Long-lived *pou1f1* mutant mice (Snell dwarf) and long-lived *ghr* KO mice both have constitutively elevated CMA in the liver, when tested as young adults [34]. CMA degrades CIP2A, a positive regulator of MYC abundance, and thus CMA indirectly reduces MYC levels by reducing CIP2A [23]. Mice hemizygous for the *myc* gene are long-lived [35]. Snell dwarf mice, which have elevated CMA, have reduced protein levels of CIP2A and MYC in liver, kidney, and skeletal muscle, without mRNA changes [34], suggesting one possible connection between the elevation of CMA in Snell dwarf mice and their exceptional longevity [34, 35].

A two-pronged proteomics approach combining lysosomal targetomics and whole-liver proteomics provided evidence that CMA negatively regulates the abundance of ACLY and ACSS2, the two enzymes responsible for cytoplasmic acetyl-coA synthesis, in the livers of young adult *ghr KO* mice [36]. These proteins are highly sensitive to CMA activity in NIH3T3 and AML12 cells [36]. Loss of function in either ACLY or ACSS2 is sufficient to extend lifespan in *Drosophila* [37–39]. While the role of ACLY and ACSS2 in mammalian longevity is not yet defined, some data suggest that small molecule inhibitors of ACLY may mimic the metabolic effects of calorie restriction, a well-characterized method for extending mouse lifespan [40].

We have now evaluated both sexes of the UM-HET3 mouse stock for age-related changes in CMA. UM-HET3 mice are derived from a four way cross of inbred mouse strains, making each animal genetically unique [41, 42]. We found no evidence of an age-related decline in CMA or critical CMA proteins in these mice. To the contrary, we found that LAMP2A increases with age in the livers of UM-HET3 mice. In isolated liver lysosomes and in some tissues, we identified significant effects of sex on CMA or the expression of key CMA proteins. Most of the previously published studies on CMA use only one sex of mice or rats, usually males. Thus, important effects of sex on CMA might exist in other mouse and rat stocks, but remain undetected.

## RESULTS AND DISCUSSION

### Age does not decrease LAMP2A levels in UM-HET3 or C57BL/6J mice

**Figure 1:**
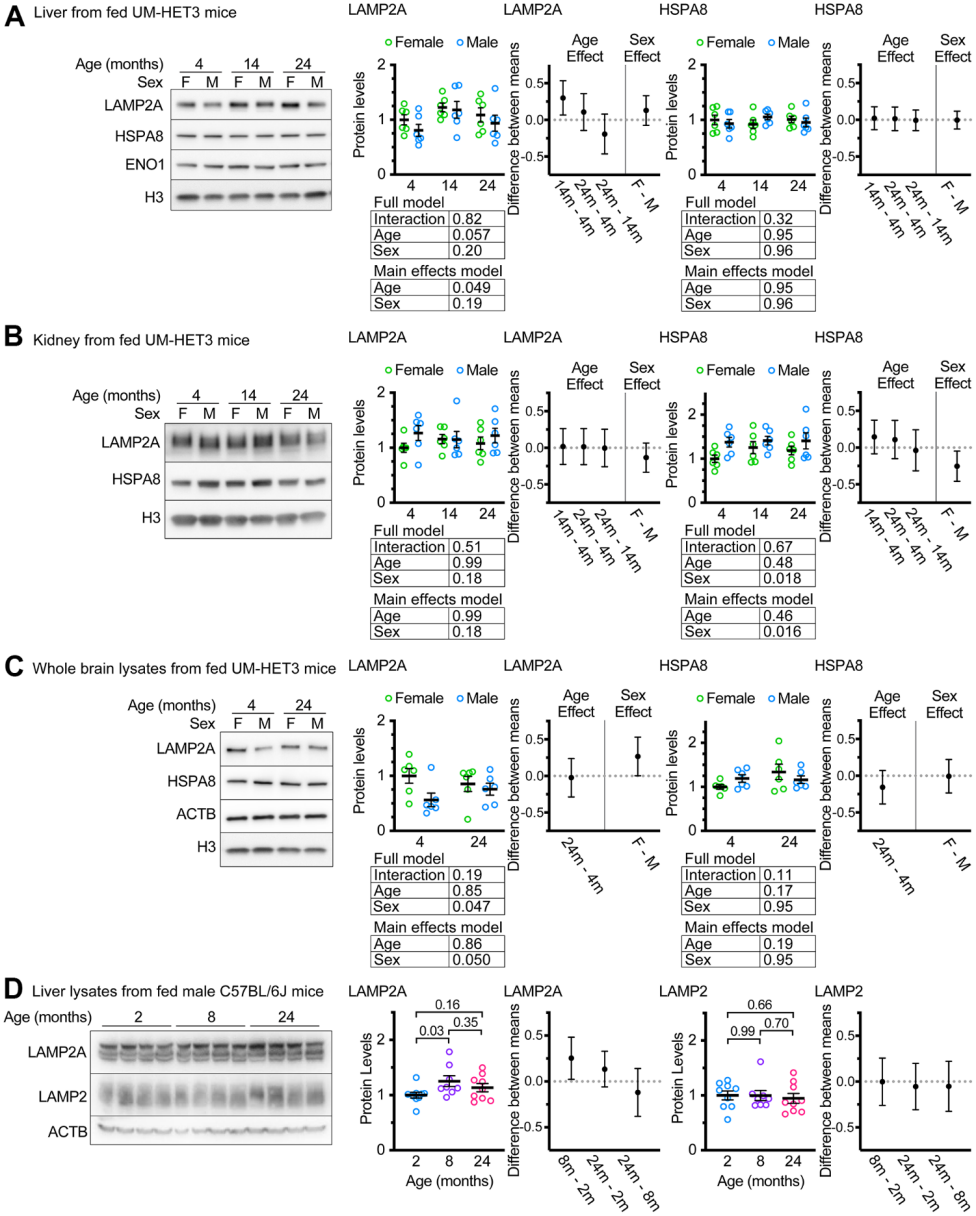
**Age does not decrease LAMP2A levels in UM-HET3 or C57BL/6J mice.** (**A**) Representative western blots and quantifications of LAMP2A and HSPA8 are shown in whole livers lysates from ad libitum fed male and female UM-HET3 mice of ages 4, 14, and 24 months. H3 and ENO1 are loading controls. n = 6 animals per group. (**B**) Representative western blots and quantifications of LAMP2A and HSPA8 are shown in whole kidney lysates from ad libitum fed male and female UM-HET3 mice of ages 4, 14, and 24 months. H3 is a loading control. n = 6 animals per group. (**C**) Representative western blots and quantifications of LAMP2A and HSPA8 are shown in whole brain lysates from ad libitum fed male and female UM-HET3 mice of ages 4, and 24 months. H3 and ACTB are loading controls. n = 6 animals per group. (**D**) Representative western blots and quantifications of LAMP2A and total LAMP2 are shown in whole livers lysates from ad libitum fed male C57BL/6J mice of ages 2, 8, and 24 months. ACTB is a loading control. n = 9 for 2- and 24-month groups, n = 8 for 8-month group. Statistical analysis was performed in GraphPad Prism 9. Lines are drawn at each mean, with error bars showing S.E.M. p-values derived from 2-way ANOVAs of both “full models” and “main effects models” are shown beneath each graph. “Estimation plots” are shown to the right of each graph; error bars on estimation plots show the 95% C.I. for the difference between the means of the indicated groups. p values displayed directly on the graphs are derived from unpaired t tests.

Whole liver lysates from male FVB mice at 22 months of age have been reported to have sharp reductions in LAMP2A levels compared to samples derived from 6-month-old animals [31]. We assessed whole liver lysates from ad libitum fed male and female mice of ages 4, 14, and 24 months. There was a marginally significant effect of age on whole liver LAMP2A levels (p = 0.049 by a main-effects model 2-way ANOVA; the interaction term was not significant in the full model). However, contrary to previous reports using inbred strains of rodents [30, 31], UM-HET3 mice at ages 14 and 24 months appeared to have more LAMP2A, on average, than mice at age 4 months (Figure 1A), and the increase between 4 and 14 months was statistically significant. There was no effect of age on HSPA8 (the chaperone that selects proteins for degradation by CMA) levels in the liver of UM-HET3 mice (Figure 1A).

CMA has been implicated in the pathogenesis of age-associated diseases in the kidney and brain [1, 43, 44]. We measured LAMP2A and HSPA8 levels in the kidneys of ad libitum fed UM-HET3 mice at ages 4, 14, and 24 months. There was no evidence of an age-related effect on the abundance of these proteins. However, males had significantly more HSPA8, when normalized to H3 levels, than females (Figure 1B). We also measured LAMP2A and HSPA8 levels in whole brain lysates from ad libitum fed 4- and 24-month-old UM-HET3 mice. There was no effect of age on either LAMP2A or HSPA8. However, males had significantly less LAMP2A in the brain than females (Figure 1C).

An age-related increase in LAMP2A levels has never been reported for liver of mice or rats. We considered the possibility that this finding might be specific to UM-HET3 mice. Several previous studies have identified changes in CMA with age in male C57BL/6J mice [18, 33, 45]. We evaluated the levels of LAMP2A and total LAMP2 in the livers of male C57BL/6J mice at ages 2, 8, and 24 months (Figure 1D). We found that livers from C57BL/6J mice at 8 months of age had significantly more LAMP2A than livers from 2-month-old animals. However, there were no significant differences between any of the other age groups. There were no differences in total LAMP2 between any of the age groups.

Even though male Fisher-344 rats [30] and male FVB mice [31], have age-related declines in LAMP2A in the liver, our data suggest that male C57BL/6J and male (and female) UM-HET3 mice do not have an age-related decrease in LAMP2A in the liver. Because an assessment of LAMP2A protein levels at the level of the whole tissue is not sufficient to draw conclusions about the status of chaperone-mediated autophagy, we proceeded to an evaluation of lysosome enriched fractions.

### Age does not cause a decrease in lysosomal LAMP2A levels in UM-HET3 mice

**Figure 2:**
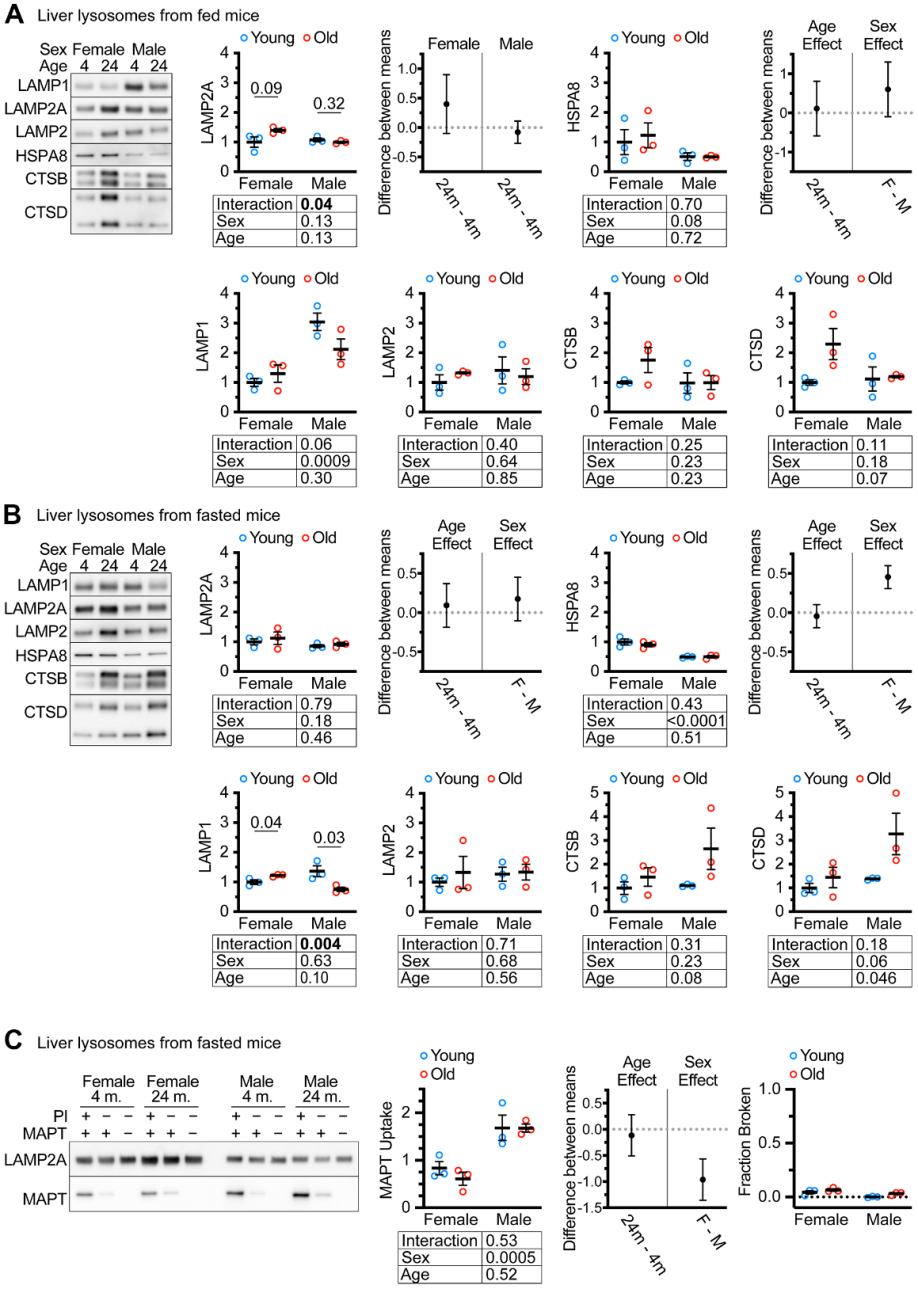
**CMA differs by sex, but not by age in UM-HET3 mouse liver lysosomes.** (**A**) Representative western blots and quantifications are shown for the indicated proteins in 4 μg of the light (CMA+) lysosome fraction from the livers of ad libitum fed male and female mice of ages 4 and 24 months. (**B**) Representative western blots and quantifications are shown for the indicated proteins in 4 μg of the light (CMA+) lysosome fraction from the livers of male and female mice of ages 4 and 24 months that were fasted for 18 hours before euthanasia. (**C**) Representative western blots and quantifications are shown for a substrate binding and uptake assay using the light (CMA+) lysosome fraction from male and female mice of ages 4 and 24 months that were fasted for 18 hours before euthanasia. The right panel shows the fraction of broken lysosomes. In each case, fewer than 10% of lysosomes were broken. n = 3 for each group in every experiment. Statistical analysis was performed in GraphPad Prism 9. Lines are drawn at each mean, with error bars showing S.E.M. p-values derived from 2-way ANOVAs are shown beneath each graph. “Estimation plots” are shown to the right graphs for LAMP2A and HSPA8 (the two proteins most important for CMA activity) Error bars on estimation plots show the 95% C.I. for the difference between the means of the indicated groups. p values displayed directly on the graphs are derived from unpaired t tests.

Previous studies have reported the isolation of “CMA+” and “CMA-” lysosome subpopulations from the livers of mice and rats using metrizamide-based discontinuous density gradients [46]. The light “CMA+” lysosome fraction is enriched in MTORC2 component RICTOR, and the heavy “CMA-” lysosome fraction is enriched in the MTORC1 component RAPTOR [47]. We used Histodenz-based gradients to prepare subcellular fractions of light “CMA+” and heavy “CMA-” lysosomes from the livers of ad libitum fed female and male UM-HET3 mice of ages 4 and 24 months. These gradients separated lysosomes into fractions enriched for RICTOR (CMA+) and RAPTOR (CMA-), at the expected densities, similar to previous studies using metrizamide gradients (Supplementary Figure 1). Because the light lysosome fraction is thought to be responsible for most CMA activity [46, 47], we evaluated this fraction for the abundance of key CMA proteins. We analyzed 4 μg of protein for each sample by western blot, finding no age-related change in LAMP2A, HSPA8, or total LAMP2 (Figure 2A). Previous studies have reported age-related increases in cathepsins in liver lysosomes [30]. However, the changes in the abundances of CTSB and CTSD did not reach significance in our experiments (although p = 0.07 for CTSD) (Figure 2A).

Many CMA studies examine CMA after prolonged fasting. We fasted both male and female UM-HET3 mice of ages 4 and 24 months for 18 hours and assessed CMA+ lysosomes for changes in lysosomal markers. We found no age-related decrease in either LAMP2A, HSPA8, or total LAMP2 (Figure 2B). In fasted mice, the age-related increase in CTSD reached statistical significance. Surprisingly, there was an age-related increase in LAMP1 in females and an age-related decrease in males, with a sex x age interaction effect at p = 0.004 (Figure 2B).

### CMA substrate uptake is not changed by age in lysosome enriched fractions from UMHET3 mice

Lysosomes from fasted mouse livers have high CMA activity that can be measured by *in vitro* CMA substrate uptake assays [46]. Here, “uptake” is defined as the difference between the amount of CMA substrate MAPT present in lysosomes treated with protease inhibitors (PI; to block degradation) and the amount of MAPT present in lysosomes without protease inhibitor treatment. We found that age did not affect the uptake of MAPT, but lysosomes from males had significantly higher MAPT uptake than lysosomes from females (Figure 2C). Fewer than 10% of lysosomes were determined to be broken by hexosaminidase latency test [48], meeting the technical criterion for the uptake assay (Figure 2C).

### Age does not modify the effects of fasting on CMA-sensitive proteins in UM-HET3 livers

**Figure 3:**
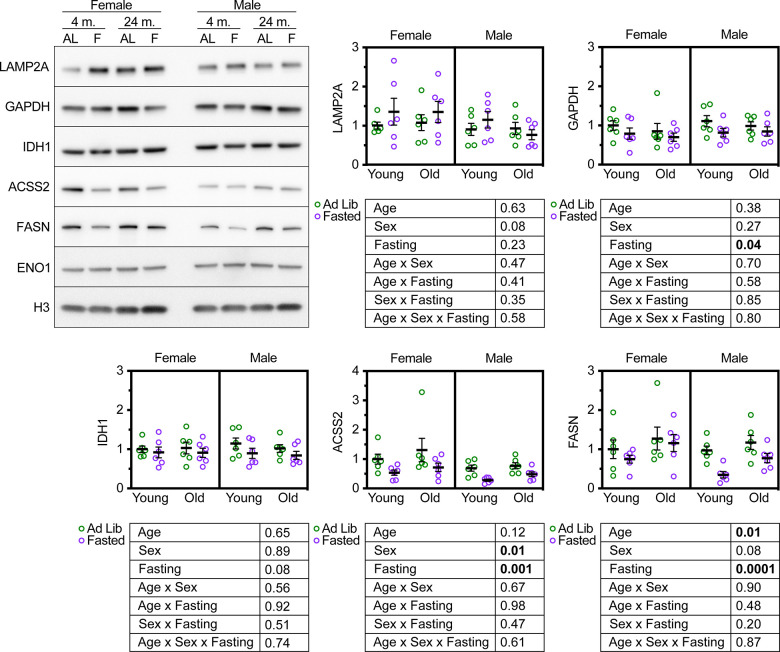
**Age does not modify the effect of fasting on CMA target protein abundance in UM-HET3 liver.** Representative western blots and quantifications are shown for the indicated proteins in whole liver lysates from male and female UM-HET3 mice of ages 4 and 24 months. Mice were either fed ad libitum (AL; green circles) or fasted (F; purple circles) for 18 hours prior to euthanasia. n = 6 for every group. Age was not found to modify the effects of fasting on CMA-sensitive proteins, by 3-way ANOVA. Statistical analysis was performed in GraphPad Prism 9. p-values derived from 3-way ANOVAs are shown beneath each graph.

Mice deficient for LAMP2A in the liver have an elevation in the abundance of several proteins involved in glycolysis, such as GAPDH [22]. We have shown that CMA is both necessary and sufficient to regulate the abundance of several proteins essential for cytoplasmic acetyl-coA generation and fatty acid synthesis, including IDH1, ACSS2, and FASN [22, 36]. *ghr* KO mice and *pou1f1* mutant (Snell dwarf) mice, both of which are long-lived, have constitutively active CMA, and have decreased liver levels of IDH1, ACSS2, and FASN [36]. We measured the abundance of GAPDH, IDH1, ACSS2, and FASN in the livers of male and female UMHET3 mice at 4 and 24 months. The mice were either fed ad libitum or fasted for 18 hours prior to tissue sample collection. We reasoned that if aging reduces CMA, then there should be an increase in the relative abundance of these four CMA-sensitive proteins in the livers of old mice, relative to young controls. Aging caused a small, but significant increase in FASN, but did not affect the abundance of the other CMA sensitive proteins (Figure 3).

CMA is activated by fasting [20]. We reasoned that these CMA-sensitive proteins should decrease in the livers of UM-HET3 mice in response to fasting-induced CMA activation. If CMA decreases with age, then it might be the case that age will blunt the effects of fasting on the abundance of CMA-sensitive proteins. We found that fasting significantly reduced the abundance of GAPDH, ACSS2, and FASN (as predicted), and caused a near-significant reduction in IDH1 (p = 0.08) in UM-HET3 liver (Figure 3). However, age did not modify the effects of fasting on the abundance of CMA sensitive proteins (Figure 3). This result is consistent with the finding that lysosomes from 4- and 24-month-old UM-HET3 mice have equal CMA substrate uptake activity.

### Concluding remarks

This paper examined four parameters pertaining to CMA in both sexes of mice in young and old animals, (1) whole tissue levels of key CMA proteins LAMP2A and HSPA8, in liver, kidney, and brain, (2) liver lysosome levels of LAMP2A and HSPA8, (3) lysosomal substrate uptake in isolated liver lysosomes from fasted mice, and (4) changes in levels of CMA-sensitive proteins in mouse liver, in response to fasting. All of these assays provide only limited information into changes in CMA with age, and none of them monitor CMA activity *in vivo*. However, the assays used in this paper were very similar to the assays that yielded the original evidence for a change in CMA with age [30, 31, 46]. CMA activity *in vivo* can now be measured directly in mice expressing a fluorescently labeled CMA substrate, that accumulates on lysosomes when CMA is active [45]. Sophisticated proteomics analyses can also be employed to gather highly detailed information on the lysosomal uptake of hundreds or thousands of endogenous CMA substrates [36]. However, these expensive and labor-intensive experiments are difficult to justify since the commonly used battery of initial experiments yielded no evidence supporting the hypothesis that CMA changes with age in the mouse models used by the labs that contributed to this study.

Previous studies examining changes in LAMP2A levels, CMA substrate uptake activity, or the abundance of CMA-sensitive proteins with age, have entirely relied upon the use of isogenic mouse or rat strains [15, 31, 46]. Recent studies comparing related stocks of genetically different mice have clearly demonstrated that results obtained from any single isogenic rodent stock cannot safely be generalized to other isogenic or inbred stocks, or to the species in general [49–52], because results observed in any one such stock (such as inbred C57BL/6) are frequently not replicated in other stocks, even when tested in the same laboratory at the same time. UM-HET3 mice are the four-way cross stock used by the NIA Interventions Testing Program, and for a recent study mapping the genetics of longevity [53]. In UM-HET3 mice, we found no evidence for an age-related decrease in CMA. To the contrary, we found evidence of increased LAMP2A expression in the liver with age.

The differences in results between this work, and other works identifying an age-dependent decrease in LAMP2A, might arise because of differences between the particular genetic stocks of mice or rats used in these studies. However, because we did not successfully replicate an age-dependent decrease in LAMP2A in any tissues of the two mouse stocks we examined, we must acknowledge that the difference between our results and the previous studies could also arise from idiosyncrasies of the husbandry conditions used, such as temperature, proximity of other stocks, olfactory or sonic environment, organic or inorganic impurities in water, or vivarium microbiota. Composition of the chow and composition of the bedding and environmental enrichment could also contribute to the differences between our work and that of others.

The current assumption that age-related decline in LAMP2A contributes to the pathogenesis of many forms of late-life disease now seems less likely to be generalizable to all mouse stocks and all vivaria. It is possible that in certain stocks and colonies an age-related decline in LAMP2A or CMA in one or more tissues could contribute to a specific form of age-dependent disease, but a general assumption that CMA declines with age no longer seems warranted.

There is extensive literature showing organ-specific sexual dimorphisms in macroautophagy and lysosomal gene expression in mice and rats [54]. These sex differences in macroautophagy are especially relevant to the progression of cancer and diseases of the heart and nervous system [54–56]. Despite the known effects of biological sex on macroautophagy, effects of sex on CMA have been largely ignored, because most CMA studies use only a single sex of mice [31, 46], or represent experiments performed in cultured cells, where the sex of the donor is not taken into account [57, 58]. Knockout of the *lamp2a* gene in Medaka (Japanese rice fish) leads to a female-specific dysregulation of carbohydrate metabolism in the liver [59], similar to what is observed in mice with a liver-specific *lamp2a* deletion [22]. This finding suggests that CMA might have sex-specific roles on regulating protein expression in Medaka liver. The results of our study suggest that sex affects CMA in a tissue-specific manner. While it is not the goal of this study to characterize the mechanisms that underlie the sex differences in CMA, future studies characterizing these mechanisms might prove to be very important for the ongoing efforts to develop CMA-enhancing therapies. All future studies of CMA in vertebrate models should include an analysis of both sexes. Sex differences in CMA might modify other CMA-related phenotypes in important ways that could be missed in studies using only a single sex of mice.

## MATERIALS AND METHODS

### Antibodies

Commercially available antibodies used at University of Michigan were acquired as follows: ACSS2 (CST: 3658S; rabbit host; Lot: 2), ACTB/β-Actin (CST: 8457L; rabbit host; Lot: 7), CTSB/Cathepsin B (CST: 31718S; rabbit host; Lot 1), CTSD/Cathepsin D (Abcam: 75852; rabbit host; Lot: GR260148-28), ENO1 (CST: 3810S; rabbit host; Lot: 2; KO validated), FASN (Abcam: 22759; rabbit host; Lot: GR3192402-1; KO validated), GAPDH (CST: 2118S; rabbit host; Lot: 14), H3 (Abcam: 176842; rabbit host; Lot: GR3277361-2), HSPA8/Hsc70 (Abcam: 154415; rabbit host; Lot: GR307969-3), IDH1 (Abcam: 172964; rabbit host; Lot: GR130705-18; KO validated), LAMP1 (Abcam: 24170; rabbit host; Lot:GR3255586-1), LAMP2 (Invitrogen: MA5-17861; rat host; Lot: WC3211254), LAMP2A (Abcam: 125068; rabbit host; Lot: GR23784-34), MAPT/Tau (CST: 46687S; rabbit host; Lot: 1), RAPTOR (CST: 2280S; rabbit host; Lot: 13), RICTOR (CST: 2114S; rabbit host; Lot: 7), TUBA (CST: 2144S; rabbit host; Lot: 6). The antibody for LAMP2A (Abcam: 125068) was validated by siRNA knockdown in our previous manuscripts [36, 60].

Commercially available antibodies used at Calico were acquired as follows: LAMP2A (Abcam, ab18528, lot# GR3265250-2), LAMP2 (Abcam, ab13524, lot# GR3245901-11), ACTB/β-actin (Cell Signaling, 8457S, lot# 7).

### Lysosome isolation

Mice were dissected at approximately the same time for each experiment (between 9 and 10 AM, with the dark period ending at 6 AM).

For mice collected in the ad libitum condition, the mice were allowed free access to food until the time of humane euthanasia. Upon dissection, mice were qualitatively assessed for the presence of food in the stomach (and all mice used in the study had food in the stomach). For mice collected in the “fasted” condition, mice were placed in a clean cage with no food 18 hours prior to dissection. All mice were allowed free access to water, until the time of humane euthanasia.

For CMA substrate uptake assays, isolated lysosomes were incubated in uptake assay buffer: 300 mM sucrose, 10 mM MOPS, pH 7.2, 10 mM ATP (Sigma, A26209), 10 mg/mL recombinant HSPA8 (Abcam, ab78431). Recombinant Tau (Sino Biological, 10058-H07E), and/or protease inhibitors (Sigma, 11836153001) were added, as indicated on the figure panels. Lysosomes were incubated at 37° C for 20 minutes, pelleted, and then washed with 300 mM sucrose, 10 mM MOPS, pH 7.2, and then prepared for analysis by western blotting.

To assess lysosomal breakage, lysosomes were diluted in uptake assay buffer: 300 mM sucrose, 10 mM MOPS (pH 7.2), and 10 mM ATP, and treated to the same conditions as the lysosomes used in the substrate uptake assay, in parallel to that experiment. At the end, the lysosomes were pelleted (no washes) and the supernatant was collected. The pellets and supernatants were assessed for hexosaminidase activity using a 4-nitrophenyl-N-acetyl-b-D-glucosaminide colorimetric assay, as described by [48].

### Mouse stocks and husbandry

All animal experiments conducted at the University of Michigan were approved by the University of Michigan Institutional Animal Care and Use Committee. Mice were housed in Specific Pathogen Free facilities, with sentinel animals checked quarterly for infection (all tests were negative). Mice had free access to food (5L0D, Lab Diet: 0067138) and water until the start of the experiments, unless otherwise stated. The mice were maintained on a 12-hour light, 12-hour dark cycle, with lights-on starting at 6:00 AM. All mice were euthanized between 9:00 AM and 10:00 AM.

The genetically heterogeneous UM-HET3 mouse stock was produced as previously described [41, 42]. Briefly, F1 hybrid CByB6F1/J (JAX stock #100009) females are crossed to F1 hybrid C3D2F1/J (JAX stock #100004) males. The CByB6F1/J mothers are generated by crossing BALB/cByJ females to C57BL/6J males. The C3D2F1/J fathers are generated by crossing C3H/HeJ females to DBA/2J males. All test animals are the offspring of these two F1 hybrid stocks.

Animal experiments conducted at Calico were approved by the IACUC at Calico. For C57BL/6J male mice housed at the Calico vivarium, animals were also maintained on a regular 12-hour light, 12-hour dark cycle and had free access to regular mouse chow and drinking water. Sentinel animals were screened at a monthly basis to monitor and ensure that the environment was free of common, known pathogens.

### Statistical analysis

Statistical analyses and graph generation were performed with GraphPad Prism 9. Results of 3-way and 2-way ANOVAs are reported directly on the figures or in the figure legends.

## Supplementary Material

Supplementary Figure 1
