# Supplementary material for: LAMP2A, and other chaperone-mediated autophagy related proteins, do not decline with age in genetically heterogeneous UM-HET3 mice
Source: Aging (Albany NY). 2023 Jun 13;15(11):4685–98. doi: 10.18632/aging.204796 (PMC10292871; doi:10.18632/aging.204796)
Supplement: Supplementary Figure 1 [file aging-15-204796-s001.pdf]

SUPPLEMENTARY FIGURE

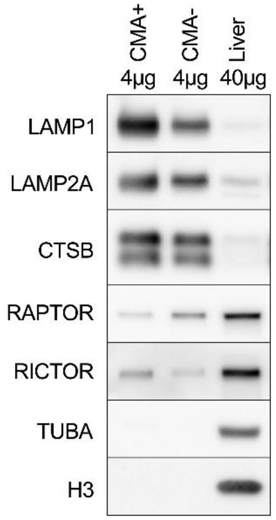

Supplementary Figure 1. Data in support of Figure 2 – controls for lysosome enriched fractions. 4 µg of light (CMA+), 4 µg of heavy (CMA-), or 40 µg of whole liver lysates were analyzed for LAMP1 (lysosome membrane), LAMP2A (lysosome membrane), CTSB (lysosome matrix), RAPTOR (CMA- lysosomes), RICTOR (CMA+ lysosomes), TUBA (cytoplasm), and H3 (nucleus). The representative western blot indicates appropriate separation of CMA+ and CMA- lysosomes, similar to what has been observed by previously published methods.
